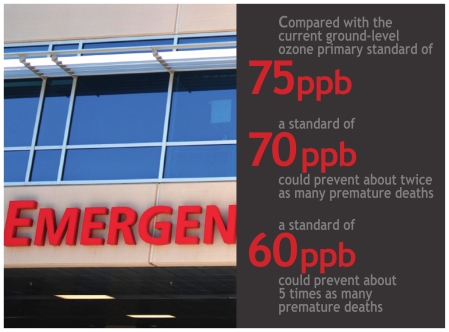# POLICY:EPA’s Ground-Level Ozone Standard Redux

**DOI:** 10.1289/ehp.118-a115

**Published:** 2010-03

**Authors:** Bob Weinhold

**Affiliations:** **Bob Weinhold**, MA, has covered environmental health issues for numerous outlets since 1996. He is a member of the Society of Environmental Journalists

The U.S. Environmental Protection Agency (EPA) is taking another crack at setting National Ambient Air Quality Standards for ground-level ozone, which EPA administrator Lisa Jackson says is “one of the most persistent and widespread pollutants we face.” In March 2008, the agency revised the standards for the first time in 11 years, far longer than the 5-year interval stipulated in the Clean Air Act. This time, the interval is far shorter, and many industry and state officials are legally challenging the EPA’s authority to reconsider the standard so quickly. But in announcing the proposed rule making, the agency contended it’s authorized to do so because the 2008 standards aren’t grounded in science and don’t sufficiently protect public health or the environment, as required by the Clean Air Act.

According to the EPA, adverse health effects from elevated airborne ozone include premature death from heart or lung disease, reduced lung function, increased susceptibility to respiratory infections, and increased hospital admissions, emergency department and doctor visits, medication use, and school absences. Ground-level ozone is a by-product of atmospheric reactions between nitrogen oxides, volatile organic compounds, methane, and carbon monoxide in the presence of sunlight. The precursor chemicals typically come from combustion processes, industrial and vehicle emissions, chemical solvents, and natural sources.

The 2008 primary standard—which is designed to protect public health—was set at 75 ppb, although a 60- to 70-ppb range had been unanimously recommended by the agency’s Clean Air Scientific Advisory Committee (CASAC). The secondary standard—which is designed to protect the environment—was in the same form and concentration as the primary standard, addressing just peak exposures, not cumulative exposures as CASAC had recommended. [For more information, see “Ozone Nation: EPA Standard Panned by the People,” *EHP* 116:A302–A305 (2008).]

On 7 January 2010 the EPA announced it was proposing a primary standard within the range of 60–70 ppb along with a CASAC-sanctioned secondary standard within the range of 7–15 ppm-hours (a unit that accounts for both concentration and length of exposure to that concentration). According to EPA calculations published as a supplement to the March 2008 regulatory impact analysis, a primary standard of 70 ppb would, by the year 2020, prevent about twice as many premature deaths and nearly that many nonfatal heart attacks as a standard of 75 ppb, and would prevent more than 2.5 times as many hospital and emergency room visits and missed work and school days. A primary standard of 60 ppb, compared with 75 ppb, would prevent about 5 times as many premature deaths, about 4 times as many nonfatal heart attacks, about 8 times as many hospital and emergency room visits, and 9 times as many missed work and school days.

Just over 21% of the 3,141 U.S. counties have an ozone monitor. About 60% of the U.S. population lives in monitored counties that exceed a 70-ppb standard, and about 67% live in monitored counties that exceed a 60-ppb standard, according to data from the EPA and the U.S. Census Bureau. The percentage living in nonattainment areas could be substantially higher when the EPA makes its final determination, tentatively planned for July 2011, of which counties violate its new standards. This determination is based on the 3-year average of the fourth highest reading over an 8-hour period, and includes consideration of factors such as estimates of ozone concentrations in unmonitored counties. On the other hand, says Janice Nolen, assistant vice president for national policy and advocacy at the American Lung Association (ALA), ongoing reductions in ozone precursors are projected to lower ozone levels in the future.

The World Health Organization recommends a health standard of 51 ppb, which appeals to Norman Edelman, chief medical officer for the ALA. “If you want to set a level that’s safer for everyone, 51 is better,” he says. But he says the ALA recognizes that only 6 of the 675 monitored counties would currently meet that standard. Instead, the ALA continues to push for the lowest end of the range recommended by the CASAC. “If we can get to 60, that’ll be a huge step forward,” Nolen says.

The EPA is legally prohibited from considering implementation costs when setting its standards. However, many business and government officials are highlighting costs as they lobby the agency. Calli Barker Schmidt, director of environmental communications for the National Association of Home Builders, says the home building industry may not be required to take substantial direct action to reduce ozone generation, but any construction moratoriums that may be imposed on counties that don’t meet the standards could be costly for her clients. “It’s like if someone misbehaves in class, then everyone has to stay inside for recess,” she says.

Howard Feldman, director of regulatory and scientific affairs for the American Petroleum Institute, says, “We call it moving the goal posts in the middle of the game. This is very real and very costly. It’s going to have a major societal impact.”

The 60-day public comment period closes 22 March 2010, and the EPA plans to announce its final standards by 31 August 2010.

## Figures and Tables

**Figure:**